# Properties and Functions of Myochondrocytes and Myochondroblasts in Different Human Cartilage Tissues—An Overview

**DOI:** 10.3390/cells14191504

**Published:** 2025-09-26

**Authors:** Ctibor Povýšil, Radim Kaňa, Martin Horák, Martin Kaňa

**Affiliations:** 1 Institute of Pathology, First Faculty of Medicine and General University Hospital in Prague, 116 36 Prague, Czech Republic; ctibor.povysil@lf1.cuni.cz; 2 Institute of Postgradute Studies, Charles University, 116 36 Prague, Czech Republic; 3 Department of Otorhinolaryngology, Head and Neck Surgery, General University Hospital, 128 08 Prague, Czech Republic; radim.kana@vfn.cz; 4 Department of Radiology, Homolka Hospital, 150 30 Prague, Czech Republic; martin.horak@homolka.cz; 5 Department of Otorhinolaryngology, Head and Neck Surgery, First Faculty of Medicine and University Hospital Motol, Charles University, 116 36 Prague, Czech Republic

**Keywords:** α-smooth muscle actin (SMA), myochondroblasts and myochondrocytes, cartilage heterogeneity, auricular cartilage, articular cartilage, cartilagineous tumours, regeneration, cartilage transplantation, transdifferentiation

## Abstract

A subset of chondrocytes in various human cartilage tissues, including neoplastic, regenerative, and normal cartilage, expresses α-smooth muscle actin (α-SMA), a protein typically found in smooth muscle cells. These α-SMA-containing chondrocytes, termed myochondrocytes and myochondroblasts, may play important roles in cartilage physiology, regeneration, and structural integrity, particularly in auricular and articular cartilage. This review synthesizes current knowledge regarding the terminology, distribution, and biological significance of these cells across normal, osteoarthritic, transplanted, and neoplastic cartilage. We summarize key findings from immunohistochemical studies using markers such as S-100, α-SMA, and SOX9, along with ultrastructural confirmation of myofilament bundles via electron microscopy. Current evidence suggests that myochondrocytes exhibit enhanced regenerative potential and contribute to matrix remodeling. Furthermore, their presence reflects the inherent cellular heterogeneity of cartilage, potentially arising from transdifferentiation processes involving fibroblasts, mesenchymal stem cells, or chondroblasts. Finally, TGF-β1 and PDGF-BB are identified as a critical modulator of α-SMA expression and chondrocyte phenotype. A deeper understanding of nature and function of myochondrocytes and myochondroblasts may improve interpretations of cartilage pathology and inform strategies for tissue engineering and cartilage repair. This review highlights the need for further investigation into the molecular regulation and functional roles of these cells in both physiological and pathological contexts.

## 1. Introduction

Actin isoforms are essential components of the eukaryotic cytoskeleton. Among them, α-smooth muscle actin (α-SMA) functions as a contractile isoform and is predominantly expressed in smooth muscle cells, as well as in myofibroblasts, myoepithelial cells, and pericytes. By contrast, β-actin and γ-actin are typically cytoplasmic isoforms in non-muscle cells [1,2,3,4]. Unexpectedly, α-SMA-positive cells have also been identified in cartilage, including in cartilage neoplasms [5,6,7,8], and subsequently in normal articular cartilage [9,10,11], damaged articular cartilage [11,12,13], and auricular cartilage [14,15]. These cells are likewise observed during tissue remodeling during healing processes or following autologous chondrocyte transplantation [11,13,16,17]. In this review we consolidate evidence on α-SMA-positive chondrocytes and chondroblasts, herein referred to as myochondrocytes and myochondroblasts, respectively. We summarize their terminology, distribution, cytological features, and putative functions, integrating data from immunohistochemistry (e.g., S-100, α-SMA, SOX9), ultrastructure, and cell culture models. The available literature indicates that a distinct subset of cartilage cells express α-SMA and may contribute to cartilage physiology, matrix remodeling, and repair [18,19,20]. Our own studies have described two related α-SMA-positive cell types: myochondroblasts, identified in benign chondroblastoma and characterized by abundant cytoplasm, lobulated nuclei, and peripheral bundles of dense microfilaments [5,6,21]; and myochondrocytes, which occur in normal and osteoarthritic cartilage and contain irregularly oriented microfilament bundles [11,13,14,15,16,17]. Together, these observations support the concept that α-SMA-positive cartilage cells represent a specialized chondrocyte subpopulation relevant to both physiological tissue maintenance and pathological remodeling.

Despite growing interest, systematic in vivo studies of human myochondrocytes remain limited. Multiple extrinsic and intrinsic factors, including growth factors, vitamins, and hormones, are known to influence chondrocyte proliferation and phenotype in vivo and in vitro [20]. Notably, α-SMA expression confers enhanced contractile potential to stress fibers (SFs), as established for myofibroblasts [3,4]. However, myochondroblasts and myochondrocytes differ from myofibroblasts in immunophenotype, ultrastructure, and genetic features [5,6,7,8,14,16,19,22]. Taken as a whole, understanding the origin, regulation, and roles of these α-SMA-positive cells may refine diagnostic interpretation in cartilage pathology and inform strategies for tissue engineering and repair.

Representative histological and ultrastructural figures included in this review are original and unpublished images from the authors’ archives. They are presented solely as illustrative examples of findings previously described in our published studies and do not represent new data.

## 2. Human Cartilages Containing Myochondrocytes

### 2.1. History of the Description of Cartilage Cells with Myofilaments in Human Cartilage Tissue

**Figure 1:**
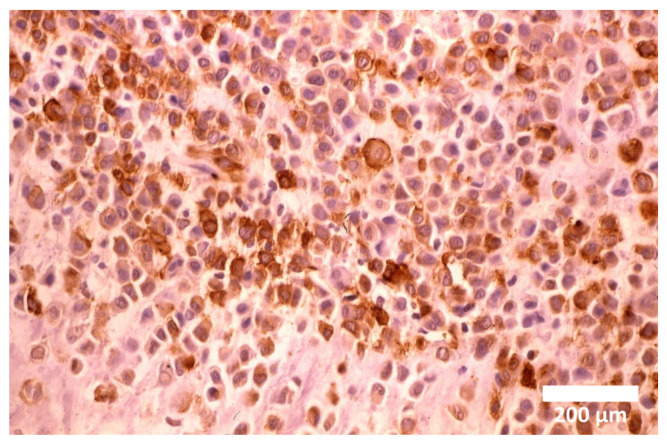
Diffuse cytoplasmic immunopositivity of α-SMA in myochondroblasts of benign chondroblastoma. Magnification ×100.

**Figure 2:**
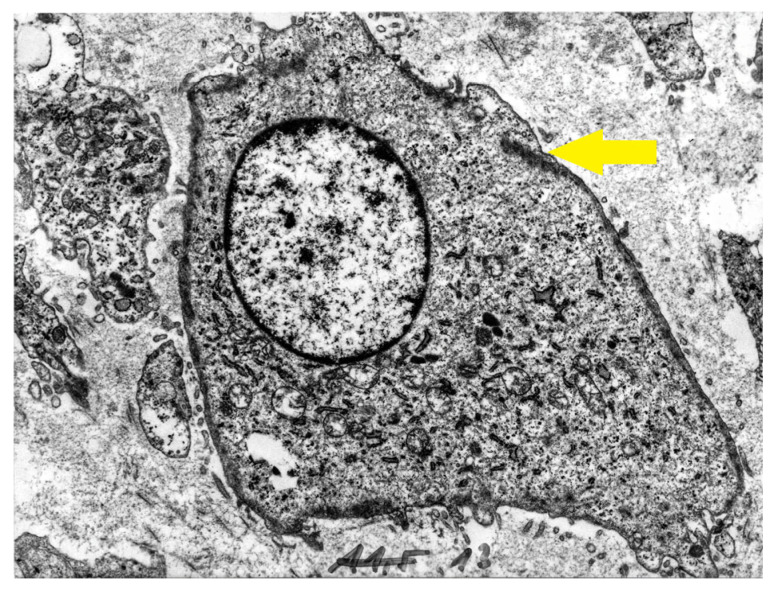
Electron micrograph of a neoplastic myochondroblast with cytoplasmic processes containing bundles of dense microfilaments located near the plasma membrane (yellow arrow). Original magnification ×4000.

**Figure 3:**
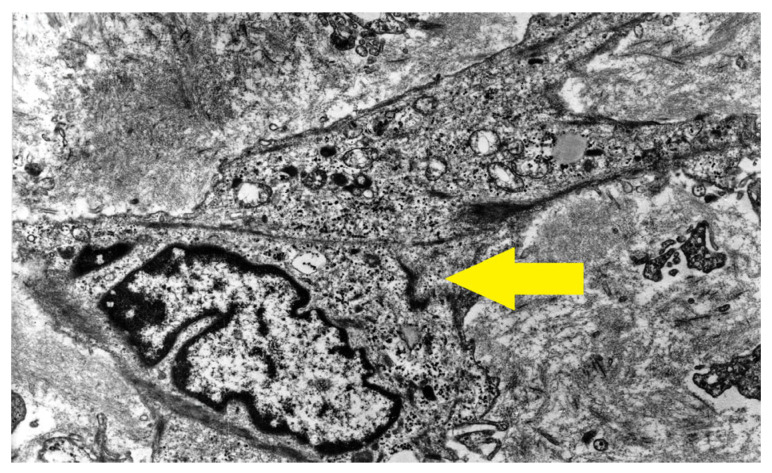
Electron micrograph of a neoplastic myochondrocyte containing irregularly oriented bundles of microfilaments in the cytoplasm (yellow arrow). Original magnification ×6000.

The first description and definition of human α-smooth muscle actin (SMA) containing chondroblasts and chondrocytes, herein termed myochondroblasts and myochondrocytes, originated from our preliminary report on primary human chondrogenic bone tumors, specifically benign chondroblastoma [5]. Two years later, our findings (Figure 1, Figure 2 and Figure 3) were published with the same terminology in Human Pathology journal [6], accompanied by detailed electron microscopy characterization of these cells (Figure 2 and Figure 3). Different features between myochondroblast (Figure 2) and myochondrocyte (Figure 3) are demonstrated on these electron microscopic pictures. In that study, we also reported myochondroblasts in three cases of chondromyxoid fibroma and in the myxoid areas of three cases of chondrosarcoma. In an earlier ultrastructural study [21], we had observed microfibrillar intracytoplasmic material in chondroblasts, but these structures could not be immunohistochemically characterized at that time [6,7].

Hasegawa et al. [8] briefly reported SMA positivity in some chondrogenic tumors in their immunohistochemical study; however, their reports lacked descriptions and picture documentation of some characteristic immunohistochemical and ultrastructural features of these cells. Loty et al. [22] observed rat chondrocytes containing α-SMA in their cytoplasm after fluorescence labeling when cultured on bioactive glass ceramic, later describing similar findings under different experimental conditions. B. Eyden [7] documented many instances of well-developed smooth muscle myofilaments in human non-muscle cells of various origins. He also cited our work interpreting this process as “transdifferentiation toward smooth muscle cells”.

Nielsen et al. [23] confirmed our earlier results identifying similar cells in chondroblastoma and chondromyxoid fibroma of bone and adopted our terminology. We were the first to propose the terms myochondroblasts and myochondrocytes [5,6]. However, A. C. Kim and C Spector, M. [9], while confirming our findings, questioned the necessity of the “myo-” prefix. Although the term “α-SMA containing chondrocytes” is also valid and acceptable, the widespread use of the term “myofibroblast” suggests that similar nomenclature is generally well-received. Romeo et al. [24] demonstrated partial myofibroblastic differentiation in chondromyxoid fibroma, driven by TGF-β1 signaling. This terminology has since been used in subsequent studies. Our subsequent immunohistochemical and electron microscopy investigations revealed numerous distinctive features of myochondrocytes across different cartilage tissues, enhancing our understanding of these still-enigmatic cells [11,13,14,15,16,17]. Later studies confirmed the α-SMA gene expression in human and myochondrocytes under various in vivo and in vitro conditions [19,20,25]. Additionally, microRNAs have been shown to play a significant role in regulating gene expression during cell differentiation [19].

Animal chondrocytes cultured on different substrates have been extensively studied, yielding important insights. Earlier studies on the chondrocyte cytoskeleton [25,26,27,28,29] laid the groundwork for understanding this phenomenon. α-SMA-positive cells in monolayer cultures were shown to express the α-SMA gene [26,27]. Similar findings were reported in pellet cultures of human mesenchymal stem cells exposed to TGF-β1 [19,30]. These observations are consistent with earlier studies describing α-SMA-positive cells in human osteoarthritic cartilage [9,10,11,31,32], where isolated cells continued to express the α-SMA gene [10].

### 2.2. Normal Human Cartilages Containing Myochondrocytes

#### 2.2.1. Auricular Cartilage

It is now recognized that myochondrocytes play a crucial physiological role in auricular cartilage tissue. The mature lamellae of adult auricular cartilage exhibit a tri-lamellar histological structure [14,15]. The intercellular matrix of the central zone contains a large number of elastic fibers, whereas these fibers are present in only small amounts in the peripheral layers. Chondrocytes in the central layer do not exhibit α-SMA positivity. In contrast, the most metabolically active cells, likely myochondrocytes, are located in the external layers adjacent to the perichondrium and display α-SMA expression. This suggests that normal cartilage tissue contains two subsets of cartilage cells—chondrocytes and myochondrocytes—that differ in their functions. Similar findings published Zhang and Spector [33].

**Figure 4:**
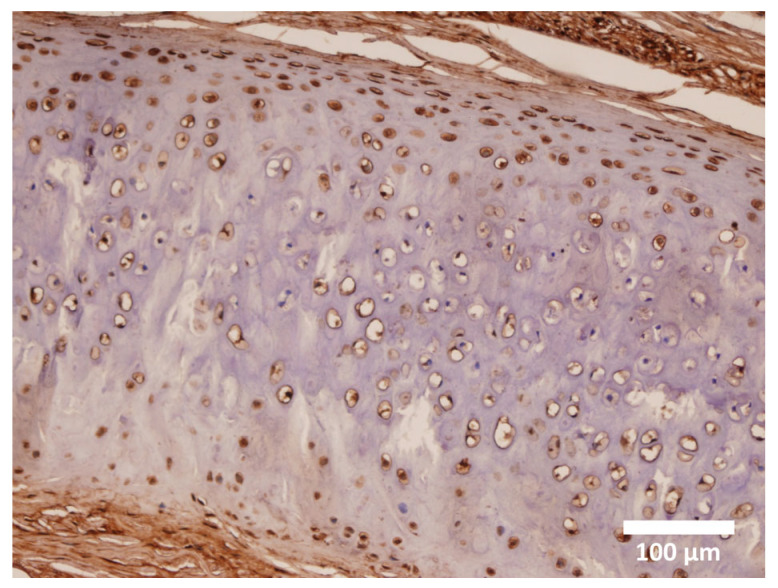
Details of adult elastic auricular cartilage showing myochondrocytes predominantly located in the peripheral zones of the cartilage. Magnification 200×.

A notable and previously unreported finding is the variation in density and shape of α-SMA and S-100 protein-positive chondrocytes on the external surface of the convex sides of auricular cartilage. We hypothesize that the distribution of myochondrocytes in these external regions, particularly in areas of pronounced curvature such as the convex side of the pinna, is essential for maintaining the characteristic shape of human auricular cartilage [15]. RT-PCR analysis [14] confirmed the presence of α-SMA in auricular chondrocytes, consistent with the immunohistochemical findings (Figure 4). However, other tissue-specific isoforms of actin, such as α-SKA, α-CAA, and γ-SMA, were not detected [14].

#### 2.2.2. Myochondrocytes in Normal Articular Cartilage

Another type of chondrogenic tissue that typically contains a small number of myochondrocytes is normal articular cartilage. This was first described by Kim A. C. and Spector M. [9], in cartilage from arthritic joints. We obtained comparable results in our study of the non-arthritic, i.e., normal articular cartilage [11]. Immunohistochemical analysis using monoclonal antibodies for α-SMA was conducted on human normal articular cartilage samples obtained during total hip replacement surgery for femoral neck fractures in patients without osteoarthritis symptoms. Approximately 20% of chondrocytes in the superficial region of normal articular cartilage expressed α-SMA [11], whereas fewer α-SMA-positive cells were found in the deeper regions.

Similar cells have been described in humans [34,35,36] and bovine [35] meniscus, and canine intervertebral discs [37]. The fibrocartilage of the meniscus is composed of a dense network of type I collagen with relatively low proteoglycan content, conferring high tensile strength. Our findings confirm the previous published observations [33] demonstrating the presence of relatively frequent myochondrocytes in this type of cartilage. These results are also consistent with our experience in routine diagnostic pathology.

### 2.3. Myochondrocytes in Pathologically Changed Articular Cartilage

#### 2.3.1. Osteoarthritic Cartilage

**Figure 5:**
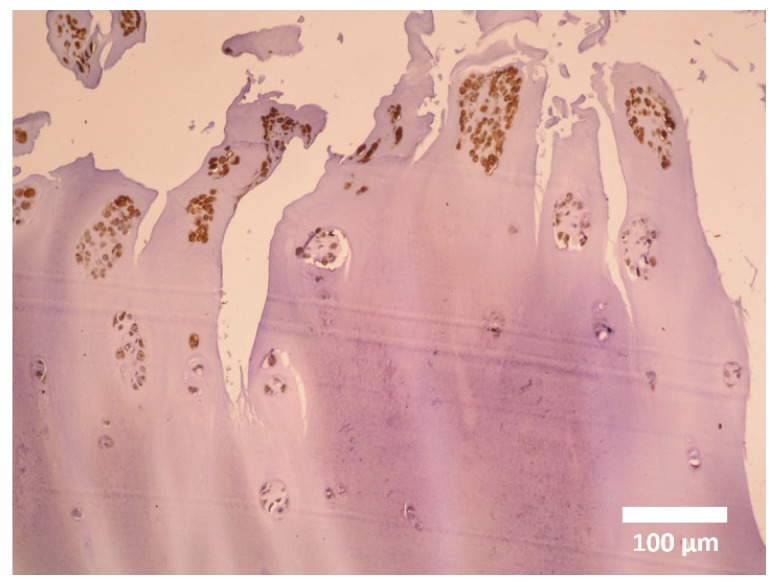
Surface of the femoral head articular cartilage from an osteoarthritic patient. The chondrocytes forming clusters stained positively for α-SMA (×200).

Osteoarthritis is a progressive disease that can lead to considerable disability. Degenerative changes in articular cartilage are evident through both macroscopic and microscopic examination, manifesting as superficial fibrillation, cartilage fragmentation, and decreased thickness [11]. These injuries trigger repair responses, which are associated with structural changes [31]. Several factors, such as growth factors, vitamins, and hormones, influence the proliferation and differentiation of chondrocytes during the disease process [31]. In osteoarthritic cartilage from femoral heads and knees of our hemophilic patients with arthritic changes, we observed myochondrocytes forming clonal groupings (Figure 5) in neighborhood of the cartilage defects [11,13]. These cells can be visualized using antibodies against α- SMA [11,13]. Prior to our work, A. C. Kim and M. Spector [9] had described these cells in osteoarthritic cartilage. It has been suggested that these cells may alter their normal behavior and contribute to matrix degradation and abnormal extracellular matrix production, as hypothesized by Sherwood (2019) [31]. MicroRNA profiling of osteoarthritic cartilage compared with normal cartilage revealed a 16-microRNA osteoarthritis gene signature [31].

In post-traumatic cartilage defects, removed before cartilage transplantation in young patients, myochondrocytes also predominated [11,13,17]. Ten months after transplantation, the newly formed cartilage displayed a mixture of partly hyaline cartilage and fibrocartilage with myochondrocytes [11,17]. The new cartilage tissue exhibited incomplete maturation, lacking zonal formation [11]. However, the majority of the cartilage cells expressed α-SMA. RT-PCR analysis confirmed the presence of α-SMA, as well as β- and γ-actin in all specimens [11].

These findings indicate that myochondrocytes in hyaline cartilage, apart from those in the surface layer of normal tissue of this type, are strongly associated with osteoarthritic changes and predominantly emerge during the reparative process [9,11,13]. Our observations align with those of A. C. Kim and M. Spector [9], though their study focused exclusively on material from arthritic joints. In their subsequent work [12], also described α-SMA-containing chondrocytes during the healing of surgically created defects in adult canine articular cartilage. Additionally, our previous study demonstrated the presence of desmin and α-SMA containing chondrocytes in human defective articular cartilage [13]. Similar cells have been observed in dysfunctional temporomandibular joint discs [38].

At the molecular level, it has been suggested that a significant proportion of adult articular chondrocytes begin to re-express a chondroprogenitor phenotype during osteoarthritic degeneration [31]. These findings highlight the diverse adaptation mechanisms of chondrocytes, allowing for the transformation of cartilage immunophenotypes in response to different conditions. Chondrocytes likely activate smooth muscle features as part of the healing process.

#### 2.3.2. Autologous Chondrocyte Transplantation of Two Different-Seeded Materials

Autologous cartilage is a valuable transplant material widely used in various clinical fields. Interestingly, we observed a predominance of α-SMA spindle-shaped cells in cultures of chondrocytes (Hyalograft C) used for transplantation in the treatment of post-traumatic articular cartilage defects [16,17]. These cells did not resemble typical chondrocytes, either in their shape or immunophenotype, as S-100 protein-positive prechondrocytes were absent. Brno chondrograft culture contained round cells showing features of differentiated myochondrocytes expressing S-100 protein and α-SMA. In contrast, in the case of Hyalograft C was made up of a fibrillar network composed of biomaterial fibers of the esters of the hyaluronic acid and the cells seeded onto these fibers and resembled fibroblasts and myofibroblasts and expressed only α-SMA [16,17]. No signs of chondrocyte differentiation were observed and so we supposed they may represent pre-chondrocytes or immature mesenchymal cells.

## 3. Discussion

Actins are ubiquitous eukaryotic proteins found not only in muscle cells but also in various other cell types across different histogenetic origins. They play critical roles in cellular functions such as muscle contraction, maintenance of cellular shape and integrity, and other processes [39]. In non-muscle cells, approximately half of the cytoplasmic actin exists as monomers (G-actin), which are ATP-bound. The other half is polymerized into actin filaments (F-actin), visible under electron microscopy [6,7] and immunohistochemistry [39] as stress fibers (SFs). These filaments aggregate with myosin [39,40] to form larger bundles. F-actin is a dynamic polymerized structure that can depolymerize back into G-actin monomers. Studies of chondrocyte cultures demonstrate that repeated passaging induces so-called dedifferentiation of chondrocytes, which is associated with actin polymerization and the formation of SFs [9,39,40]. Dedifferentiated chondrocytes acquire an amoeboid or fibroblast-like shape, and contain prominent, irregularly distributed SFs [20,39,40]. SFs, composed of F-actin bundles crosslinked by α-actinin and other proteins, often incorporate myosin [7,20]. The formation of SFs significantly alters chondrocyte morphology [20]. By contrast, differentiated chondrocytes are rounder, have a cortical F-actin ring with diffuse punctate actin staining, and lack SFs [31]. Chondrocyte phenotype is regulated by multiple extracellular stimuli and intracellular signaling pathways [7,26,27,28,41]. Schofield et al. [41] demonstrated that TPM3.1 inhibition causes F-actin reorganization from SFs back to cortical F-actin in passaged chondrocytes. Several authors propose that the actin cytoskeleton acts as a key regulator of chondrocyte dedifferentiation [26,27,28,41,42]. Electrophoresis can differentiate six isoforms of actin in vertebrates, divided into classes [39,42]. The first class includes ubiquitous cytoplasmic β-actin and γ-actin. The remaining four isoforms are tissue specific, present in skeletal muscle (α skeletal muscle), cardiac muscle (α cardiac actin), and vascular and gastrointestinal muscle (α and γ-SMA). α-SMA is most commonly found in smooth muscle cells, where it facilitates contraction. In vascular smooth muscle, α actin predominates, while γ-actin is more abundant in gastrointestinal smooth muscle [42,43]. In addition to smooth muscle, α-SMA is also expressed in myofibroblasts, myoepithelial cells, pericytes [1,2,3,4] and both neoplastic [5,6,8] and non-neoplastic myochondrocytes [11,14]. It is important to note that α-SMA positive cells were present in only a limited portion of our chondroblastoma cases as well as in some other cartilage neoplasm [6,8].

At the time of these discoveries, the biological significance of myochondrocytes and myochondroblasts remained unclear. However, we excluded their classification as myofibroblasts, as they co-express S-100 protein and α-SMA, as confirmed by double-labeling immunohistochemistry [5,6]. Moreover, electron microscopy revealed no characteristic features of myofibroblasts [1,2,3,4,6]. Myochondrocytes and myochondroblasts lacked the basal lamina and micro-tendons typical of myofibroblasts. Instead, these tumor cells displayed round or lobulated nuclei, and produced multiple cytoplasmic microvilli, characteristic of chondroblasts [5,6]. The intercellular matrix of these cells contained thin filaments and fine proteoglycan granules, further supporting their cartilaginous origin [6].

Chondrocyte differentiation is a multistep process characterized by changes in cell morphology and gene expression [19]. A notable subset of chondrocytes express α-SMA in healthy, diseased, neoplastic, and regenerated cartilage. These cells, termed myochondrocytes and myochondroblasts, are identified primarily through S-100 protein, α-SMA [5,6], and SOX9 [4] labeling. The nomenclature is appropriate by analogy to myofibroblasts, a widely accepted term for contractile fibroblasts [2,3]. Myochondrocytes and myochondroblasts are well defined, distinct cell types, that differ in immunophenotype and ultrastructure [5,6,18]. Chondrocyte differentiation is regulated by multiple extracellular and intracellular signaling pathways [37,42,43,44,45], with cell shape, and cytoskeletal organization playing key roles [26,27,28]. Studies have emphasized the important role of actin organization in phenotype modulation [44], as chondrocytes cultured in a 2D monolayers acquire contractile features and exhibit increased actin polymerization [42]. They develop a contractile phenotype regulated by the actin MRTF-A-serum signaling axis [42]. Such contractile chondrocytes may participate in tissue contraction [42,46]. Importantly, Schofield et al. demonstrated that TPM3.1 inhibition or siRNA knockdown reorganizes actin filaments from SFs to cortical F-actin while increasing the G/F-actin ratio, indicating that stress fiber formation suppresses the dedifferentiated phenotype in cultured chondrocytes [41].

Although the molecular mechanisms linking chondrocyte differentiation and actin organization remain poorly understood, transdifferentiation is likely a key process [47]. Lauer et al. [20] demonstrated that extracellular stimuli and intracellular signaling pathways modulate chondrocyte phenotype during serial passaging in culture. Pro-inflammatory cytokines and growth factors play significant roles in regulating actin dynamics [21]. In culture, differentiated chondrocytes [33] display a cortical F-actin ring consistent with our electron microscopy findings in neoplastic myochondroblasts [6]. In contrast, dedifferentiated, fibroblast-like chondrocytes and S-100 protein-positive cells contain prominent SFs distributed throughout the cytoplasm [25,39,41,45]. Zaleskas et al. [48] showed that α-SMA expression can be regulated by specific growth factors. TGF- β1 upregulated α-SMA expression, while PDGF-BB downregulated it. Moreover, these factors influenced cell contractility, establishing a direct relationship between α-SMA expression and contractile potential.

Chondrocytes isolated from cartilage tissue dedifferentiate in a 2D microenvironment and lose cartilage-specific ECM expression. Transfer of these cells into an artificial monolayer culture induces a shift ingene expression, including increased type I collagen and reduced type II collagen, which is normally specific to hyaline cartilage. Subsequent redifferentiation in a 3D culture microenvironment leads to the formation of spheroidal aggregates, although this process is serum-dependent [45]. The transcription factor SOX9 is expressed early in chondrocyte differentiation.

The precise function of myochondrocytes remains incompletely understood. The presence of myochondrocytes in the superficial layer of normal articular cartilage suggests their involvement in maintaining cartilage integrity [9,10,11]. The contractile properties of α-SMA may play a role in remodeling of the extracellular matrix. It is also possible that myochondrocytes have a higher potential for regenerating compared to α-SMA negative chondrocytes. Degenerative changes in articular osteoarthritic cartilage and in hemophilic patients are associated with clonal clusters of myochondrocytes near cartilage defects (Figure 4) [9,11]. Our findings suggest that human articular cartilage retains limited regenerative potential [11]. We also observed myochondrocytes in residual cartilage removed from post-traumatic defects before transplantation [16,17].

In our studies, we compared the histological and immunohistochemical profiles of two different chondrocyte-seeded biomaterials and evaluated the outcomes of their transplantation [16]. Arthroscopic assessment at 10 months postoperatively revealed similar outcomes with both biomaterials, including the presence of numerous myochondrocytes in the regenerated cartilage [16]. This newly formed cartilage contained both S-100 protein and α-SMA-positive chondrocytes, suggesting the presence of chondrogenic progenitor cells [11]. In contrast, increased proliferation of these cells was more pronounced in various pathological conditions including osteoarthritic cartilage and in tissue following implantation of articular chondrocytes [11,16].

Our work has demonstrated that, under normal conditions, myochondrocytes are the dominant cell type in auricular cartilage [14,15], whereas in articular cartilage they are typically confined to the superficial zone [9,11]. This layered organization directly determinates the mechanical properties of auricular cartilage [15]. We identified myochondrocytes in both peripheral layers adjacent to the perichondrium, emphasizing the significance of this specialized structure. Such knowledge is particularly important for clinical applications, including auricular reconstructions and during the reconstruction of the nasal framework. Notably, the healing outcomes of elastic cartilage grafts differ significantly from those of hyaline cartilage grafts.

In chondrogenic tumors, the heterogeneous composition of neoplastic cell populations [5,6,8] reflects tumor heterogeneity [47,49]. This phenomenon occurs both, between tumors [inter-tumor heterogeneity], due to genetic and non-genetic factors, and within individual tumors [intra-tumor heterogeneity], where cells from the same tumor may exhibit distinct markers [47,49]. However, such heterogeneity may complicate the differential diagnosis of actin-positive mesenchymal tumors. Similar heterogeneity is also observed in non-neoplastic cartilage tissues, such as articular cartilage [9,11] and auricular cartilage [14,15] as well as in diseased cartilage [9,11,12,40,44] and transplanted articular cartilage [11,16,17]. Furthermore, intra-tumor heterogeneity applies not only to tumor cells but also to components of their microenvironments [47,50].

The heterogeneity observed in chondrocyte populations across cartilage types may result from a restricted transdifferentiation [47] between chondrocytes and chondroblasts, and myochondrocytes and myochondroblasts. Myochondroblasts may also develop directly from reprogrammed fibroblasts or mesenchymal stem cells [45,47]. Transdifferentiation involves genetic reprogramming [19]. Cota et al. [44] demonstrated that JAK inhibition promotes direct transdifferentiation of murine embryonic fibroblasts into chondroblasts. Similarly, pellet cultures of human mesenchymal stem cells treated with TGF-β1, showed increased α- SMA and type II collagen expression, indicating the origin of myochondrocytes through transdifferentiation from fibroblasts [44]. Chondrogenic differentiation from mesenchymal stem cells (MSCs) is regulated by multiple factors, including oxygen tension [28]. MSCs are multipotent cells capable of differentiating into osteogenic, chondrogenic, adipogenic, and myogenic lineages [50], with differentiation promoted by lineage-specific transcription factors. Heterogeneity in pluripotent stem cells may also arise during long-term embryonic stem cell culture [50,51].

Recently, Deroyers et al. [52] showed that cell migration-inducing protein (CEMIP) regulates transdifferentiation of chondrocytes into “chondro-myo-fibroblasts” expressing fibrosis markers α-SMA and type III collagen. These cells were identified in human and murine osteoarthritic cartilage but were absent in healthy cartilage, contradicting earlier reports [9,11]. Furthermore, α-SMA-expressing chondrocytes in monolayer culture demonstrated specific α SMA gene expression [31,32]. Similar findings were observed in pellet cultures of human mesenchymal stem cells treated with TGF-β1 [44,51].

Our electron microscopic studies of human bone tumors, including chondroblastoma, confirmed the presence of both myochondrocytes and myochondroblasts [3,5]. However, Aigner et al. [53] and Eyden [7] argued that the term “dedifferentiated cell” is a misnomer. We avoid this terminology, as it conflicts with the WHO classification of bone tumors [54]. Pathologists currently reserve the term “dedifferentiated” for tumors such as “dedifferentiated sarcomas”, which contain a conventional low- or intermediate-grade component (e.g., chondrosarcoma, liposarcoma) alongside a high-grade, histogenetically distinct undifferentiated component [54]. Similarly, “undifferentiated sarcomas of soft tissue” represent a heterogeneous group of spindle, round, epithelioid and pleomorphic cell tumours, that lack morphological or immunohistochemical features of specific differentiation [54].

MicroRNAs play a crucial role in regulation of gene expression during chondrocytes differentiation, acting primarily through post-transcriptional repression in both differentiated and dedifferentiated cells. Genetic analysis has revealed significant differences in microRNA expression between these two cell types [19]. MicroRNAs are therefore considered promising targets for cartilage tissue engineering and regenerative medicine [19]. In one study, microRNA expression was analyzed in differentiated and dedifferentiated chondrocytes, and real time RT-PCR was employed to validate the differentially expressed genes. Dedifferentiated chondrocytes lose their capacity to produce cartilage-specific ECM, including type II collagen and aggrecan, and synthesize type I collagen instead. The study identified 13 up-regulated and 12 down regulated microRNAs in differentiated chondrocytes compared to dedifferentiated chondrocytes. Notably, miR-491-3p, mir-140-3p, miR-140Sp, let-7dwere significantly up-regulated, showing 23.8-, 15.2-, 13.7-, and 8.7-fold increases, respectively [19].

Some of these findings align with our diagnostic pathology experience, where we observed heterogeneity in non-neoplastic, transplanted, diseased, and neoplastic cartilage tissues containing myochondrocytes and myochondroblasts interspersed with normal actin-negative chondrocytes. These observations confirm that in vitro culture models reflect many features of cartilage biology seen in patient tissues. It is likely that in future studies will uncover additional phenotypic changes in chondrocytes, further highlighting the heterogeneity of cartilage tumours. Recently, we described chondrosarcoma variant characterized by hypertrophic pericellular rings and distinct genetic alterations, which we termed, “chondrosarcoma with target-like chondrocytes” [55].

Collectively, these findings emphasize that the chondrocyte phenotype is highly plastic and can be modulated by microenvironmental stimuli and gene expression changes [19,20,44,45,48]. The mechanisms regulating α-SMA and other cytoskeletal protein expression in chondrocytes remain poorly understood, reflecting challenges in studying myofibroblasts [1,2,3]. However, comparable findings have been reported in other connective tissue cell types, including osteoblasts, fibroblasts, and fibrocartilage cells—that express α-SMA under certain in vitro and in vivo conditions [34]. The heterogeneous composition of both neoplastic and non-neoplastic chondrocyte populations likely arises from restricted transdifferentiation events coupled with genetic reprogramming [47]. Culture studies have shown that TGF-β1 induces α-SMA expression in chondrocytes, consistent with its established role in cartilage repair. Conversely, cytochalasin and PDGF-BB inhibit α-SMA expression, demonstrating that α-SMA regulation is sensitive to external factors [41,42,43,44,45]. These findings highlight the possibility of modulating chondrocyte-mediated contractility to improve the healing of cartilage injuries. Chondrocyte dedifferentiation remains a major limitation in autologous chondrocyte implantation for cartilage repair. Fortunately, studies indicate that redifferentiation of dedifferentiated chondrocytes is achievable under certain conditions, offering promise for regenerative therapies. Importantly, the histologic outcomes of elastic cartilage grafts differ significantly from those of hyaline cartilage grafts, underscoring the unique biology of each tissue type [56].

## 4. Conclusions

This review summarizes the current knowledge of myochondrocytes and myochondroblasts in human cartilage tissues. These cells play important roles in matrix remodeling, tissue regeneration, and pathologic responses and display molecular profiles distinct from conventional chondrocytes. The evidence suggests that the myochondrocyte phenotype is plastic, influenced by microenvironmental stimuli and altered gene expression, and that α-SMA expression can be modulated by specific regulatory pathways. A better understanding of the origin, regulatory mechanisms, and functional roles of myochondrocytes will not only contribute to knowledge of cartilage biology but may also inform about novel regenerative strategies and improve the diagnostic accuracy in cartilage pathology.

## Data Availability

The original contributions presented in this study are included in the article. Further inquiries can be directed to the corresponding author(s).

## Abbreviations

The following abbreviations are used in this manuscript:

αSMA	Alpha smooth muscle actin
ATP	Adenosine triphosphate
CEMIP	Cell migration-inducing protein
F-actin	Filamentous actin
G-actin	Globular actin
JAK	Cota
MSCs	Mesenchymal stem cells
OA	Osteoarthritis
RT-PCR	Reverse transcription polymerase chain reaction
SFs	Stress fibers
SMCs	Smooth muscle cells
SOX9	Box transcription factor
TGF-β1	Transforming growth factor beta 1
TPM3	Tropomyosin 3
MRTF-A	Myocardin related transcription factor
PDGF-BB	Platelet derived growth factor
